# The strength of a weak centre: pandemic politics in the European Union and the United States

**DOI:** 10.1057/s41295-023-00328-6

**Published:** 2023-03-30

**Authors:** Kate Alexander-Shaw, Joseph Ganderson, Waltraud Schelkle

**Affiliations:** 1 London School of Economics and Political Science, London, UK; 2 European University Institute, Fiesole, Italy

**Keywords:** Covid-19, Crisis response, European Union, Federalism, United States

## Abstract

The European Union presents a puzzle to political systems scholars: how can a developing polity, with all its attendant functional weaknesses, be rendered politically stable even through moments of a policy crisis? Building on insights from the literature on fiscal federalism, this article challenges much conventional wisdom on Europe’s incompleteness. This is based on the corollary of Jonathan Rodden’s concept of *Hamilton’s Paradox*: whereas a strong centre cannot resist exploitation by states because it has the means to rescue them, a weak centre’s lack of exploitable capacity may induce states to support, and even empower, it in a crisis. This article argues that in providing a contemporaneous stress-test, Covid-19 serves to expose both the pathologies of a strong-centred federation and the surprising resilience of a weak one. It highlights three polity features—powers, decision-making modes and integrity—and charts their political implications during an acute crisis. The article argues that in the EU these features incentivise cooperative ‘polity maintenance’ between polarised states, a feature absent in an American polity marked by rivalry between polarised parties. The article thus challenges notions that the EU’s incompleteness necessarily leads it to dysfunction or that it should strive to emulate established federations.

## Federal paradoxes

Crises are times when a secular process reaches a juncture at which alternatives become conceivable (Capoccia and Kelemen 2007), potentially leading to peril for political institutions. The Covid-19 pandemic has been such a moment for the European Union (EU). This incomplete polity faced an immense challenge in managing functional and political challenges through the pandemic, yet retained its political integrity in 2020–21. Meanwhile, the United States (US), despite the greater powers vested in its federal centre, experienced a deeply divisive crisis under two different presidents. Covid-19 impacted both polities simultaneously and severely. Yet curiously, the EU’s pandemic response has been no less functionally effective, and in political terms it has fared somewhat better, avoiding the politicised state-centre conflicts that have persistently characterised the American Covid experience (Rhodes 2021).^1^ This article advances the argument that the EU’s comparatively weak centre reveals distinctive strengths in an existential crisis. States in a polity with a weak centre have incentives to maintain and possibly even empower the centre, exactly because the centre does not have the holding capacity on its own. The pandemic was such a case for the EU.

The EU’s relative success in managing the disintegrative potential of the pandemic confounds the expectations of EU studies literatures tending to emphasise the polity’s immaturity and incompleteness. This article therefore sets out to develop an explanatory framework that addresses the paradoxical strength of the EU’s weak centre. Our framework builds on insight from Jonathan Rodden’s *Hamilton’s Paradox* (2005): that centralised federal power can be a weakness, since a strong centre with the means to rescue states cannot resist exploitation by them, positioning states and centre as rivals. We propose a corollary: a weak centre, with limited exploitable capacity, benefits from member states’ incentives to support and even empower in a severe crisis.

The multifaceted Covid crisis allows for the conceptualisation of strength and weakness in federal centres both in terms of economic and political resources and institutions. Inspired by the state-building tradition of Stein Rokkan et al. (1999)(see Kriesi et al, 2023, for a fuller discussion of the EU in a Rokkanian perspective), the article identifies three interrelated ‘polity features’ of the EU and US centres, which have markedly different political implications for policymaking and polity maintenance in and by member states. First, strong centres command extensive fiscal and political *powers* and resources vis-à-vis states. This creates differentiated incentives for polarisation between vertically integrated parties in the US and horizontally integrated EU member states. Second, the *decision-making mode* in the strong-centred US operates on a majoritarian calculus whereby the centre can override states, while the weak-centred EU tends to enhance the institutional veto powers of member states which states must overcome by brokering agreement. Third, polity *integrity* follows from this. In the US, the federal centre is not disciplined by the threat of secession or wholesale collapse. Even amid the extreme turmoil of President Trump’s final weeks in office, it was the democratic character rather than the fundamental continuity of the American polity that was existentially challenged (Norris 2021). In the EU, the credible background threat of polity disintegration has important conditioning effects on the behaviour of leading actors and institutions. EU leaders showed a keen awareness of the polity’s fragility. Their crisis response inverted Rodden’s federal paradox and demonstrated that from weakness emerges a stabilising strength.

Focusing on the Covid-19 crisis through 2020–2021, this article describes how these three polity features produced distinct political implications that can explain why the EU took the path of conspicuous solidarity. First, it outlines a theoretical framework, incorporating essential weak-strong fiscal federal dynamics and alternative approaches to polity formation. Second, it operationalises polity features, describing their political effects for the EU and the US. The framework is then applied in two detailed case studies, which break the pandemic down into healthcare and economic concerns, describing key state and central leaders’ public dialogues, positioning and bargaining stances. The article concludes by summarising this explanation for federal paradoxes, which rests on institutional incentives for polity maintenance.

## Polity politics beyond fiscal federalism

Fiscal federalist theory is concerned with aligning state policymakers’ incentives and central institutions to create an enduring ‘best of both’ equilibrium (Oates 2005). Key to this is balancing the powers of centres vis-à-vis states. Alexander Hamilton, the first US Treasury Secretary and an eminent federal theorist, warned that weak centres which apportion too many powers to states, risk “rendering the empire a nerveless body, incapable of regulating its own members, insecure against external dangers, and agitated with unceasing fermentations in its own bowels” (Hamilton and Madison 1787 [1864], 167). But the kind of strong federal government that Hamilton advocated is vulnerable to what Rodden (2005) called *Hamilton’s Paradox*: a centre with strong fiscal and redistributive powers is weak to resist exploitation by profligate states. State leaders understand that a strong centre must help because it can help and are incentivised to “raid the fiscal commons”, overspending to deliver constituency benefits (Oates 2005, 349). Stable federations thus must strike a balance—granting states revenue-raising capacity to encourage fiscal independence, while strictly marshalling budgets and limiting central governmental discretion to intervene. Here, the US is broadly seen as a successful case, having reached equilibrium through a series of testing adolescent crises, most notably in the 1840s (Rodden 2005).

There should be little reason to fear bailout exposure within the EU, given weak federal bargains in treaties that retained fiscal decentralisation (Rodden 2005, 279). However, the post-2010 euro-area crisis invited unfavourable comparisons with the US (Fabbrini 2013), and calls for stronger fiscal-budgetary powers for Brussels modelled on the latter (Henning and Kessler 2012; Kelemen and Teo 2014). The desired effect is typically to create instruments allowing for stabilising intergovernmental transfers and pooling government debt, while restraining moral hazard among member state leaders. A second relevant strand of EU studies scholarship is more concerned with how recent crisis dysfunctions have arisen from EU institutional incompleteness. The EU is seen as ‘failing forward’, opting for lowest common denominator policies that tackle symptoms but not the underlying cause of too little fiscal capacity (Jones et al. 2016; De Grauwe 2013, 29; Howarth and Quaglia 2021).

This article offers a rejoinder to both, while sharing these scholars’ concern that US comparisons remain valuable from an EU studies perspective, owing to “social, economic and political traits shared with the EU”, and the aforementioned fact that the US is considered a role model for a “United States of Europe” (Tortola 2014, 1353). The argument takes the EU and US as dissimilar cases of compound polities—one a union of nation-states, the other a fully articulated federation—to explain an outcome that is the opposite of what the literature would expect. As a polity, the US had a relatively bad crisis, the EU a relatively good one.

To pin down the institutional features that make for strong and weak centres, we start from Stein Rokkan’s theory of state-formation and mass politics that he combined with Albert Hirschman’s theorem of exit, voice and loyalty (Bartolini 2005, 3–47). For Rokkan et al. (1999, 100–101), these three terms were about adaptation (exit, voice) and maintenance (loyalty) of political systems under pressure. Historically, demarcating boundaries against other polities and exercising authority over this bounded territory necessitated concessions regarding political participation and some forms of social security to make people comply (Rokkan et al. 1999, 100–104; 123–131). This internal institution-building to ensure loyalty is directly conditioned by the strength of its demarcation (Ferrera 2005, 21).

The EU is unconventional in all three respects. Its external boundaries are ill-defined thanks to continuous enlargement and Brexit, and even internal boundaries can be reactivated by member states citing crisis. Its centre has uneven and shifting authority, strong in economic regulation but very weak on fiscal matters (Genschel and Jachtenfuchs 2014). At the centre, citizens’ indirect representation by national elected executives dominates direct representation in the European Parliament. This starkly contrasts with the US, an established bounded territory able to defend its borders with military means. Presidential authority allows rule by executive order, and federal macroeconomic policy resources are globally unparalleled. Pervasive majoritarian legitimation, from the President to state and local governments, creates direct voice channels up and down the polity. This was particularly important for the two political systems at hand, because democratic theory tells us that such compound polities over a vast area are characterised by the dualism of partisan and territorial politics (Lipset and Rokkan 1967, 9–11). Alignments through a vertically integrated party system, in contrast to horizontal inter-state cooperation and neighbourhood effects, are relevant for citizens’ support for crisis management (Cicchi et al. 2020).

This theory also captures why Covid-19 presented a historic challenge in both the EU and US. It called for public authority over extended territories, command over borders and the loyalty of citizens to accept intrusive measures that no democratic government had mandated in the post-war era. Citizens’ movements were curtailed and entire economies sent into hibernation. In return, states compensated businesses and workers with unprecedented largesse. Media reporting scaled up and down the size of communities with which citizens were meant to empathise. But in every respect, the EU seemed to be disadvantaged in its capacity to manage the Covid-19 pandemic. The polity is weak in terms of bounded territory, binding authority and bonds of loyalty. This is not true of the US polity.

Our aim is to explain why there is a pattern to the EU’s robustness in containing this crisis politically that is paradoxically linked to its weaknesses in terms of Rokkan’s theory. A polity’s performance during an acute crisis is not merely a function of its institutions, but the *incentives* that they create for actors to contest or cooperate, to politicise or depoliticise. The EU’s weak formal structures create strong incentives in both centre and states to maintain the polity proper, coming together to forge policy compromises that renew the widely shared sense that loyalty is preferable to exit. This can explain political representatives switching from the pursuit of national interest in normal times to polity maintenance in severe crises. No such self-preserving incentives are generated in the established US federation, with the presidential administration serving as a political rival to member states.

## Features and implications of weak and strong centres

The framework suggests that three polity features demarcate strength and weakness in the two centres. However, the political implications of these features are not straightforward. These are outlined in this section and summarised in Table 1.

**Table 1:** Features and implications of weak and strong centres

	US	EU
*Polity features*		
Powers	Core state and coercive	Regulatory and coordinative
Decision-making Mode	Majoritarian-partisan with Presidential emergency powers	Intergovernmental-executive with technocratic agenda-setting
Integrity	Stable	Fragile
*Political implications*		
Dominant Form of Polarisation	Inter-party (vertical)	Inter-state (horizontal)
Bargaining Dynamics	Competitive	Problem-solving
Expectations of Crisis Policymaking	Zero-sum	Positive/negative-sum

### Polity features

The first relevant polity feature is policy powers, formal capacities vested in institutions at the centre that determine the scope of its authority. The US federal government possesses ‘core state powers’ over defence, border control, and revenue-expenditure. US states retain discretionary over some taxes, notably sales and income, but the federal government is characteristic of modern tax states. In 2019, US federal taxes raised 16.3% of GDP in revenue against spending equivalent to 21% (CBO 2020). Such fiscal powers go far beyond the EU centre, which has no discretionary power to tax and very limited debt issuance, its revenue derived chiefly from member contributions and its ordinary spending capacity capped at around 1% of members’ gross national income (2014–20). The EU remains primarily a regulatory polity, albeit one that in some areas (state aid, competition policy) reaches beyond even the US federal government. While making inroads on select core state powers, such as border control and monetary policy, it falls far short of a federal administration (Genschel and Jachtenfuchs 2014). Importantly, in both polities, public health administration remains mostly the preserve of states. A significant difference, however, relates to the US federal government’s authority to prevent communicable diseases entering the country under the *Public Health Service Act*, a competence that remains an EU member state discretion.

Second, the US government relies on majoritarian decision-making throughout the federation, which makes for particularly strong voice channels. The US President is directly elected by an electoral college that empowers smaller states’ voters. The states also have a powerful voice in the US’ bicameral legislature via the Senate, clubbing together along partisan lines. The President makes key appointments in many federal agencies—including the Centers for Disease Control and Prevention (CDC). Constitutional checks allow elected representatives some executive constraint, increasingly leading to legislative stalemate when congressional majorities are held by different parties. By contrast, in the EU, representative democracy is firmly anchored in member states, giving way to technocratic governance at the centre. The Commission has the sole right of initiative to propose legislation to the Council for co-decision, where the voting system moderates the weight of large states and grants veto powers over treaty changes. The Parliament has now extensive co-decision authority with the Council but parliamentarians tend to represent national-territorial concerns, irrespective of partisan affiliation. In short, by design the EU does not currently approximate anything like a federal democracy (Sonnicksen 2022).

Third, and crucially, the two cases exhibit varying levels of polity integrity. The US has no legal provision for secession, the Supreme Court ruling unilateral exit unconstitutional in a precedent-setting case in 1869 (*Texas vs. White*). Secessionist parties are extremely marginal in American politics, no emancipated state has ever left the union—its integrity taken as given. By contrast, ‘secession’ from the EU, and the spectre of wider disintegration, animates a recent literature on this subject following the UK’s decision to activate the legal exit provision, Article 50 (Rosamond 2016; Schimmelfennig 2018).

Combined, these three polity features may create paradoxical effects in crisis politics. Centralised core state powers, especially when combined with direct democratic legitimation, generates contentious politics around the control of those policy capacities. The decision-making mode via parliaments or in representative bodies like the Council can amplify or attenuate underlying conflicts of ideology or state interest. The relative strength of the centre then makes integrity of the polity a more (or less) pressing issue for policymaker bargaining in polarised contexts.

### Political implications

The first implication of a strong-centred polity, both in terms of policy resources and democratic mandate, is that vertical polarisation between states and centre is likely to dominate horizontal divisions between states. While party-political polarisation in the US owes to multiple factors that cannot all be documented here, a strong centre makes state governments sensitive to its overpowering interventions, arguably reinforcing party-competitive divides. Party rivalry will be particularly intense when centre and states are not politically aligned.^2^ By contrast, when the centre is weak, lacking core state powers and direct legitimation, it is likely that partisan polarisation is attenuated by the sheer plurality of parties from all member state systems. Instead, polarisation runs along inter-state distributional lines, between net-contributors to and net-beneficiaries of a reform.

Second, we expect the majoritarian decision-making of the US centre to generate different bargaining dynamics to the EU. A strong centre can act unilaterally, using its own resources, overruling minorities among its constituent states. Its willingness to broker compromise is therefore limited, while states may have incentives to engage in open conflict with the centre to both promote their policy preferences and perhaps cultivate party careers. A weak centre must pursue cooperation, for instance via non-partisan problem-solving. While collective action problems like the joint decision trap abound in normal times, member states have incentives to collaborate in severe crises if their mutual interest in crisis containment is to be achieved (Truchlewski et al. 2021).

Finally, given a strong centre, freeriding by states on the common pool might signal inefficiency, but this does not imperil the polity’s entire existence, leaving states free to pursue their interests. This is an asymmetry in the federal paradox when we consider the EU weak centre: its limitations in terms of powers to confront a crisis can be existential. States’ responses to this threat are then constitutive. In moments of severe crisis, the awareness of central weakness may mobilise political forces among members to maintain the polity to their mutual advantage. Threatened with polity disintegration, weakness serves to refocus state minds on ‘holding together’ (Fossum and Jachtenfuchs 2017). In sum, whereas a strong centre with established fiscal and political capacities may generate zero-sum struggles to control common resources, a weak centre with shared or delegated capacities must be aware of the risk of a negative-sum outcome in which distributive conflicts call into question the viability of the whole. This animus is a recurring feature of ‘polity maintenance’ throughout the EU’s long decade of crisis leading up to Covid (Ferrera 2022).

## Case studies: Covid-19 as a polity stress-test

This section compares EU and US Covid politics through December 2021. Linking incentives to behavioural outcomes, the analysis draws on leaders’ positioning statements during influential case-within-case vignettes to illustrate state-centre dynamics at moments of heightened political tension. The analysis is split into public health and economic dimensions. The former focuses on two epicentral states, Italy and New York, which were among the first to ask the centre for help. Initial geographic concentration was a common feature in both polities but they followed quite different trajectories thereafter, with polity features explaining this divergence. As the pandemic spread, so does our case study, expanding to consider broader state-centre interactions which, in the later phases of the health crisis, were particularly focused on vaccination policy.

The economic dimension was a least-likely case for a common European response, given very weak central fiscal powers. The EU and US therefore entered the pandemic in quite different positions. Here, the case studies focus on politics at the centre, but note that this is strongly affected by prior lines of tension between states, and between states and centre. In the EU, Italy is again pivotal, given the legacy of the Euro-area crisis. In the United States, polarisation between red and blue states and the President sets the stage.

### Public health

#### European Union

**Fig. 1:**
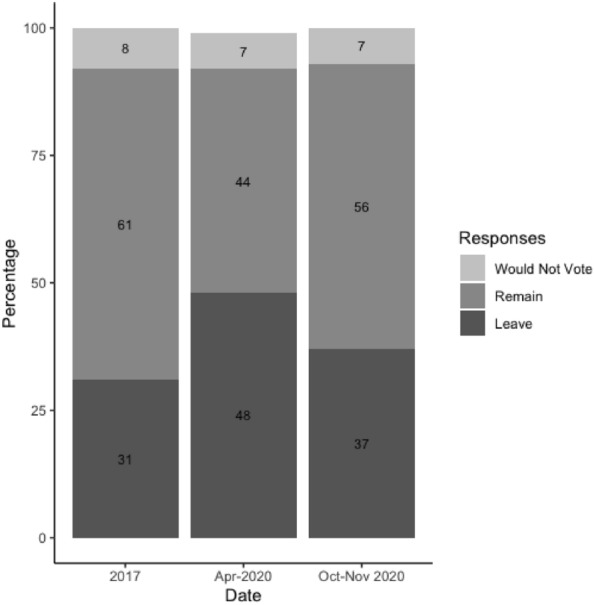
EU Membership Referendum Voting Intention—Italy. *Source*: Istituto Affari Internazionali. 2020. 2017 fieldwork month unspecified

Italy was the first western Covid-19 epicentre, its humanitarian effects materialising swiftly through March 2020, with renowned regional healthcare systems overwhelmed. Demographic, social and political-economic weaknesses combined to create a ‘perfect storm’ in the country, and authorities were surprised by the speed and escalation of contagion (Vicentini and Galanti 2021). On 28th February, Italy and the Commission activated the highest level of the EU’s Civil Protection Mechanism (CPM), a voluntary programme that marshals disaster relief across member states and through which member states can offer critical support, such as personnel or equipment. However, no member states responded, and three of four leading European manufacturers of medical protective equipment—France, Germany and the Czech Republic—imposed export controls (PIIE 2020). Beyond its immediate epidemiological threat, then, the Italian outbreak portrayed an EU either unable or unwilling to show solidarity. Polling in April 2020 found a clear majority of Italians perceiving unfair treatment and a large increase in those indicating they would vote ‘Leave’ in an EU membership referendum (Fig. 1, "Appendix"). With resentment simmering from the euro-area crisis and the Eurosceptic Five Star party largest in government, the pandemic quickly became associated with ‘Italexit’. Prime Minister Conte sounded an early warning that “the risk of [EU] failure is real” (BBC 2020).

In response, the Commission combined practical measures with public relations. In early April, President von der Leyen (2020) apologised and outlined EU support for Italy in *La Repubblica*. She rebuked other member states for forgetting that “we can only defeat this pandemic together, as a Union”, and promised that Europe would now be “rallying to Italy’s side”. The Commission used the limited powers it had, guarding against export price gouging, diverting structural funds to repatriation flights and medical procurement. CPM resources started to reach Italy in early April (European Commission 2020).^3^ Though of limited practical scope, such actions took on a symbolic significance designed to offset an image of overriding member state self-preservation.

Moving into 2021, vaccines became the key pan-EU coordination challenge. In June 2020, the EU had agreed central procurement contracts—voluntary for member states—leveraging bloc bargaining power to reduce prices and ensure equitable access for all member states. By mid-February 2021, and with the EU notably lagging behind the UK and Israel, von der Leyen admitted that this procurement effort had been imperfect: “We were late in granting authorisation. We were too optimistic about mass production. And maybe we also took for granted that the doses ordered would actually arrive on time” (Hyde 2021, 655). The EU’s AstraZeneca contract had secured comparatively low prices but apparently at the cost of priority access, ensuring suppliers facing production shortages prioritised other customers. In Germany especially, criticism of unfavourable comparisons with the UK was severe (Hyde 2021, 656). But while the Commission was blamed variously by different states for procurement naivety, this policy failure did not aggravate inter-state polarisation or spark an every-state-for-itself frenzy akin to March 2020, although Denmark and Austria were reported to be exploring authorising the non-EU authorised vaccines for domestic production (Financial Times 2021).

The lack of recriminations against the Commission was particularly notable in Italy. Given the political danger posed by the early pandemic there, it might have been a prime candidate for further politicisation, but vaccine criticism was mostly reserved for pharmaceutical companies, while the Commission and other Member States were largely absolved of blame. Prime Minister Conte criticised AstraZeneca, calling delays “unacceptable”, and Italy exploited emergency EU regulations allowing member states to seize exports if a firm was judged to be reneging on its obligations. Relative to the scale of the EU’s deal, Italy retained a small shipment (250,000 doses), and in response, member states mostly declined to criticise Europe’s weak centre, choosing instead to lend their strength to it through symbolic support of its procurement ambitions (France 24, 2021). Whereas in 2020 the EU had been incapable of compelling other states to supply Italy in its hour of need, now Italy played a leading role in shaping its post-procurement damage control strategy and defending the Commission.

As in the US, European policies aimed at increasing vaccination rates have proven controversial. Yet, vaccination rollouts have been primarily managed by member states, and laws remain the exclusive competence of national governments. Policy has varied widely, including the stick of fines (Austria) or restrictions (France) and the carrot of financial rewards (Bulgaria). But in the absence of EU powers to impose mandates, political strife has been decentralised to domestic politics and does not stick to the EU polity. This contrasts with the economic policy dimension outlined below.

#### United States

The early part of the US pandemic was also geographically concentrated, with the most acute outbreak in New York City, where cases overtook Italy’s by June 2020. President Trump declared a national emergency on 13th March, and two days later the city closed the country’s largest school system (Paumgarten 2020). The same day, state Governor Cuomo (2020) aimed a *New York Times* op-ed at Trump, urging federal action because “state and local governments alone simply do not have the capacity or resources to do what is necessary.” Specifically, Cuomo wanted federal action on expediting approvals for large-scale testing, promises of economic support for states whose economies would be hit by coming lockdowns and military deployment for emergency hospital building. Emphasising that he “[knew] the capacity of the federal government”, Cuomo applied public pressure on the centre to deploy its resources for the benefit of stricken states.

President Trump responded with a conference call in which, contra the EU’s call for solidarity, he urged state governors to take control of their own medical supply procurement. This led to clashes with Cuomo who suggested that state and federal authorities were fighting over the same consignments, bringing both levels into direct conflict (Wright 2021, 121–123). Also, unlike Conte, Cuomo had little leverage to use public opinion to concern the federal government about the breakdown of the polity. To the extent that the strong federal centre was intervening, it had effectively become a competitor to lesser-resourced states. Moreover, the federal government declined to use discretionary powers, including the Defense Production Act (DPA), which would divert private manufacturing to medical equipment. Cuomo’s public challenge to Trump echoed Conte’s plea, but the response from the centre was markedly different; where Europe had spoken in terms of solidarity and used it weak powers to the fullest, Trump was disinclined to intervene except where he saw political advantage. For example, a task force led by Trump’s son-in-law, Kushner, “turned aid to states and hospitals into a form of patronage” (Wright 2021, 125), forcing governors who wanted to protect their state into complicity. Once Trump mobilised the army corps to build hospitals, he explicitly stated he expected governors to publicly show gratitude, or else miss out on further help (ABC 2020).

The federal response is characterised by partisanship and credit claiming, with powers being deployed absent the existential necessity of solidarity gestures. While these state-centre recriminations might be attributable to the dysfunctions of the Trump administration, it was by no means limited to this. Political polarisation also affected states’ ability to muster a pandemic response, resulting in what Rocco et al (2020) have called “a patchwork of public health measures, often coloured by partisan motivations”. Furthermore, the later phase of the pandemic saw partisan polarisation continuing to undermine state-centre coordination even after President Biden radically changed tack. Biden used all federal powers, activating the DPA and prioritising vaccine rollouts, targeting 100 million doses in his first 100 days. As in Europe, a majority of citizens were vaccinated as programmes rolled out. However, vaccine hesitancy rates varied widely by partisan affiliation, with over 90% of Democrats single-dosed against 56% of Republicans through September 2021 (Gallup 2021). The federal government responded by attempting to implement a degree of coercion. On 9th September 2021, Biden signed executive orders mandating vaccination for all federal employees and contractors, with new occupational safety guidance requiring larger employers to implement either vaccine mandates or weekly testing. These federal mandates drew strong resistance from Republican governors, with at least twelve initiating legal action against the federal government over mandates (Reuters, 2021a). Inverting Trump’s laissez-faire response, the centre now found itself in open conflict with the opposing party state leaders it sought first to co-opt, then to override. Biden duly compromised, largely dropping legal compulsion in favour of educational programmes, but state and party-centre polarisation on vaccines remained high through the end of 2021.

### Economy

#### European Union

The politics of the emerging economic crisis quickly became threatening to EU integrity. The Italian epicentre was significant, as asymmetric early economic damage looked set to mirror the euro-area crisis (Celi et al. 2020), reactivating polarisation between northern and southern states. Italy was projected to see the largest GDP decline in the EU through 2020 (− 11.2%), its economy hit by Europe’s harshest lockdown measures, tourism losses and comparatively weak governance capacity (Sapir 2021). On 12th March, ECB President Lagarde announced an important functional monetary intervention, the €750bn Pandemic Emergency Purchase Programme (PEPP), but the policy was overshadowed by her remark that the ECB was “not here to close spreads” between members’ borrowing costs. An immediate clarification followed, but Italian bond spreads climbed for six days, with markets pricing in a lack of ECB commitment (Jones 2020). Led by the Netherlands, other member states signalled familiar resistance to any EU-level redistributive fiscal innovation. On 20th March, Finance Minister Hoekstra stoked inter-state polarisation, reportedly calling for a Commission investigation into southern states’ economic preparedness for the pandemic (Politico 2020a).

A bloc of nine states responded with a call for a joint debt instrument, dubbed ‘Coronabonds’ (Wilmès et al., 2020), which was justified throughout April 2020 by leaders’ public references to EU potential disintegration. Conte explicitly warned of this potential for disintegration, and called for a “strong and unified” European response to assuage the concerns of “European citizens [that] will be deeply disappointed” (Politico 2020b). This was reinforced by a rare televised intervention by Italian President Sergio Mattarella, who called on European leaders to introduce “new initiatives…to overcome old ways of thinking”, spoke of the “gravity of the threat to Europe” and reminded EU leaders that solidarity “is in the common interest” (Reuters 2020). Such veiled warnings tally with empirical evidence, as Italians appeared particularly hostile to conditionality being attached to EU financial support, with a majority preferring to exit the euro-area rather than accept such terms (Baccaro, Bremer and Neimanns,2021). The Eurosceptic Lega forced Conte to take a hard anti-ESM stance from the outset (Schelkle 2021, 48–49).

Portuguese PM Costa suggested that if agreement could not be reached, Europe’s membership and institutions would need to be reconsidered (Politico 2020c). France’s Emmanuel Macron stated*,* “if we can’t do this today, I tell you the populists will win—today, tomorrow, the day after, in Italy, in Spain, perhaps in France and elsewhere (Financial Times 2020).” The so-called Frugal Four (Austria, Denmark, Sweden and the Netherlands) favoured the existing European Stability Mechanism (ESM), with its loan conditionality, and floated modest charitable grants as an alternative. This was set out in a briefing note (Rijksoverheid 2020), delivered on the eve of the Commission proposal for a €750bn recovery fund at the end of May, which built on an earlier Franco-German proposal. While this was going on, an unconditional ESM credit line and a €100bn loan programme, SURE, fast-tracking national job retention schemes, passed without much difficulty.

Rhetorical juxtapositions of solidarity or collapse also cut through in Germany. The Grand Coalition shifted from strict ESM-adherence, to working with France in May on a third way that both avoided mutualisation and loading states with more conditional debt (Schelkle 2021). Defending the Franco-German proposal of €500bn in grants funded by the EU budget, Chancellor Merkel (Bundestag 2020, 20640) acknowledged that Covid-19 exacerbated economic inequities between states and, echoing Macron, warned of “anti-democratic forces, radical and authoritarian movements [that are] waiting for economic crises to exploit them politically.” In bringing forward their proposals, France and Germany were motivated by this political assessment of the threat posed to European integration if a common response could not be agreed. This suggests a sensitivity to the polity implications of the crisis, beyond their immediate national interests in the budget package. Bulmer (2022, 177) notes that during the early months of the pandemic, “a view began to take hold” among influential economists and within major parties that solidarity in exceptional circumstances was needed to ward off the threat of EU disintegration, with Italy the chief concern. In turn, this would secure Germany’s own industrial export-driven economy. The shift was also facilitated by Merkel’s pandemic poll bounce and underlying public preferences for the integrity of the Euro to be prioritised even above avoiding debt mutualisation (Baccaro et al. 2021). With Covid-19 representing a reckoning, this complemented the red lines in Italian public opinion charted above, although the German government stopped short of endorsing full mutualisation.

The Recovery and Resilience Facility (RRF) that emerged required Council unanimity, and as such was vulnerable to any state’s veto. At a marathon summit in late July, the Commission proposal for grants was scaled down to appease frugal members whilst maintaining sufficiently ambitious grants to appease Italy and its allies. Both sides could present the eventual deal as a win domestically (Truchlewski et al. 2021). Leaders’ orientations appeared moulded by perceptions of national self-interest and public opinion rather than party identification, reflecting the EU’s mostly horizontal polarisation structure.^4^ The outcome of the summit also seems to have been effective in containing the polity threat. Armingeon et al. (2022) find per capita member state funding allocations correlate more closely with pre-existing economic vulnerabilities, the presence of Eurosceptic sentiments and a decline in support for the EU since the start of the pandemic, than with the early severity of the pandemic. They conclude that NGEU was “an ex ante intervention to avoid another humiliating and conflict-ridden bailout” and “the political vulnerabilities that inevitably follow” (Armingeon et al 2022, 160). In this paper’s terms, this is polity maintenance in action.

By late 2020, measures of Italian EU alienation trended downwards, hinting that the RRF had the desired effect, or at least that it had not sparked further recriminations (Fig. 1, "Appendix"). In spring of 2021, the new Italian government presented a fiscal stimulus programme in two steps amounting to 4% of GDP. On 27 April, Draghi presented RRF plans to the Italian parliament and on 1st May, the Commission received spending plans worth over €190bn, €69bn in grants. The allocation was welcomed by all parties in the multi-party cabinet, with Foreign Minister Di Maio (Five Star) celebrating “an atmosphere of collaboration and enthusiasm” (Reuters 2021).

#### United States

In the US, federal institutional capacity allowed for an impressive monetary and fiscal stabilisation effort. Congress was initially united in its economic response, with bipartisan support for stimulus ensuring that the Coronavirus Aid, Relief and Economic Security (CARES) Act was passed swiftly, releasing stimulus including $500bn in direct transfers to households. The Federal Reserve directed over $2trn in liquidity support to households, businesses and local governments through 2020. The support package also included a $500bn liquidity support fund for municipal authorities, effectively backstopping states and preventing any questions over creditworthiness. These policies largely preserved local responsibility for debt, however, since liquidity support for local authorities was in the form of loans, not grants. Contrasting Europe, debt-pooling mechanisms across states did not feature, and there was no intention to equalise Covid’s revenue impact across states. Indeed, liquidity support measures for states attracted some partisan polarisation, with outgoing Treasury Secretary Steven Mnuchin declining to renew them into 2021, over the objections of the Federal Reserve.

After this initial fiscal effort, partisan polarisation around crisis policy deepened through 2020, and relationships between state governors (primarily Democratic ones) and the federal government deteriorated. In August, talks about a renewal of fiscal support measures stalled, with both parties blaming each other (Politico 2020d). Bypassing majority leaders and states, Trump signed executive orders reducing the size of support. This contributed to growing rancour between states and centre. A month later, Cuomo declared that Trump was “actively trying to kill New York City. […] they won't provide federal funding to help repair the damage from the ambush they created” (CNBC 2020).

The question of economic support for states whose balance sheets were disrupted by economic shutdowns rapidly became partisan. Republican Senate Majority Leader McConnell dismissed support for local and state governments as ‘blue state bailouts’, suggesting they consider bankruptcy. Cuomo highlighted net fiscal transfers from New York to poorer red states including McConnell’s Kentucky, declaring this “the real bailout” (Paumgarten 2020). Manifest partisanship in crisis (non-)management at the federal level and state-centre polarisation allowed for multi-level dysfunction, with interactions mired by blame-shifting.

As the pandemic wore on, space for economic consensus shrank. In December 2020, a second bipartisan stimulus bill passed Congress, but was nearly vetoed by the outgoing President, who was reluctant to boost his opponents ahead of forthcoming Senate run-offs in Georgia. When Democrats won those races, they passed the $1.9tn American Rescue Plan Act without Republican support. As in the EU recovery package, support for states was to be distributed on a formula related to the uneven economic impact of the pandemic. Unlike in Europe, this distribution was not the product of a negotiated consensus but was politically divisive, drawing criticism from Republican governors who believed they were being penalised for enacting less stringent lockdowns than Democratic states (USA Today 2021).

In late-2021, the Biden administration’s $2.2tn Build Back Better bill was blocked by West Virginia Democrat Senator Joe Manchin, who cited inflationary pressures and unfocused spending (CNN 2021). Manchin’s veto effectively reverses the Hamiltonian expectation of states calling on the strong centre to cover their losses. Rather, the majoritarian legislative process, combined with Manchin’s sensitivity to interest group and constituency pressures, led him to reject federal spending plans and abandon, rather than exploit, the fiscal commons even at the cost of bringing down the entire bill. Vertical, inter-party polarisation ensured that this was a damaging defeat for Biden, inflicted even when federal and state levels were aligned. But it has not generated an existential threat to the US polity, as a failure of a signature bill in the EU might have done if the centre had been so openly defied.

## Conclusions: polity features and crisis politics

This article identified a divergence between the weak-centred EU and the strong-centred US, during a crisis that stretched state authority to new limits. The EU performed no worse than the US in its functional handling of the pandemic (see "Appendix"), while displaying a surprising ability to manage the *political* fallout, containing its disintegrative potential through brokered institutional innovations rationalised by solidarity. Crisis events in a (horizontally) polarised EU have the potential to become existential; failure to marshal a common European response prompts fundamental questions about the merits and future stability of union. Incentives to avoid this outcome and avert conflict shaped EU leaders’ responses. As Wolff and Ladi (2020, 1025) argue, the pandemic led to “politicisation at the top with European elites [perceiving] the Covid-19 emergency as an existential threat for the EU”. After initial missteps, Covid’s destabilising potential of was quickly recognised, and became the context in which subsequent policies were mobilised (Rhodes 2021). The EU’s decision-making mode ensured that a compromise was forged which ostentatiously scaled up temporary support for the most vulnerable member states while circumventing the taboos of seizing control of national vaccine provision or imposing common debt. Such action is motivated by a logic of ‘polity maintenance’, designed to secure the loyalty of the member states and prevent them from exiting (Ferrera et al. 2021).

The US saw no equivalent collective effort that could mobilise lasting bipartisanship but relied on stronger policy powers, often utilised for partisan purposes. This tendency was made particularly visible by Trump’s clientelist style, but state-centre antagonism in the US cannot be reduced to his divisive leadership. President Biden entered office with conciliatory intent, appealing to transcendent, emergency bipartisanship. Yet, the federal vaccination campaign received hostility in certain Republican states and the Senate was split on recovery funding. No matter the leader, with the boundaries of the polity as a given and its formidable central resources to be claimed, US parties have incentives to privilege obstruction and blame. In this established polity, rivalry for the centre’s resources creates incentives for intense inter-party and state-centre polarisation, with leaders at both levels pursuing their electoral objectives via conflict. This operates as a form of political freeriding on the taken-for-granted stability of the polity. By contrast, the EU has a structural interest in projecting (if not always achieving) unity. This is not to suggest that US polarisation can be entirely attributed to federal institutions, and indeed other strong centres did not exhibit the uniquely American political dysfunctions outlined here. However, such strong configurations do not incentivise leaders setting aside differences and depoliticising a crisis, whereas the EU’s very weakness here engenders a politics of polity maintenance. The threat that a policy crisis escalates into an existential crisis of the fragile EU polity concentrates leaders’ minds on overcoming inter-state polarisation and forging compromise that can entail switching the trajectory of institutional development (Schelkle 2021).

The weakness or incompleteness of the EU centre is not without costs, which emerge in its functional vulnerability to crises. But an exploration of the surprising institutional incentives for polity maintenance does help to explain the EU’s persistence and agility in the face of daunting challenges. Indeed, the comparison between the European and the American Covid-19 experiences might give those calling for greater EU federal completeness pause for thought. The ongoing accretion of powers to the EU’s weak centre, such as redistributive and debt-raising capacity, means this depiction will not remain static. Further research should remain attentive to how increased strength in the centre might shape leaders’ behaviours and EU politics in the years and crises to come.

## Figures and Tables

**Fig. 2:**
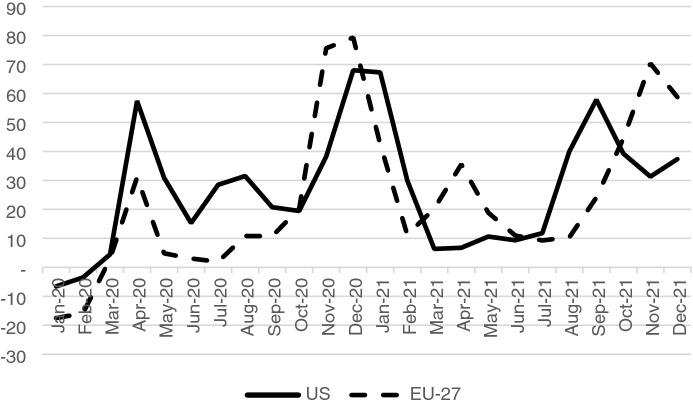
Monthly Excess Death Rates (per Million People). *Source*: Ritchie et al. (Our World in Data) (2022)

**Fig. 3:**
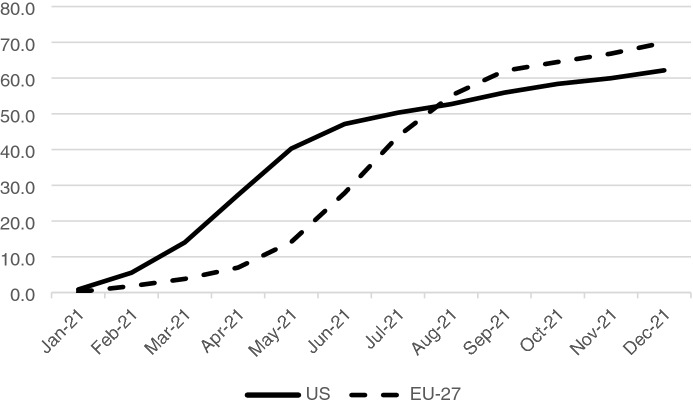
Share of Population COVID-19 Fully Vaccinated, 30 December 2021. *Source*: Ritchie et al. (Our World in Data) (2022)

**Fig. 4:**
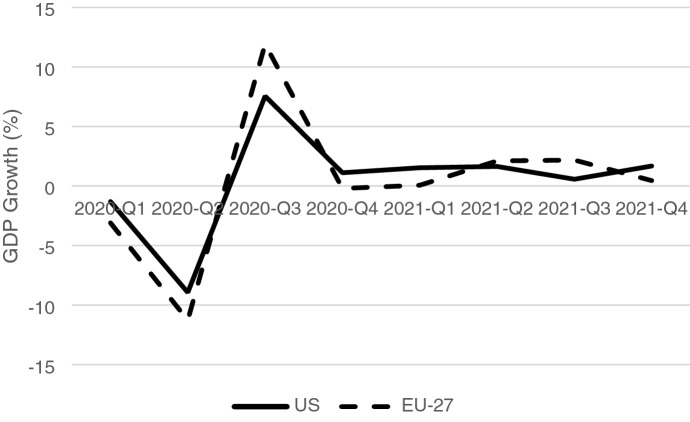
Quarterly Real GDP Growth. Source: OECD (2022)

**Fig. 5:**
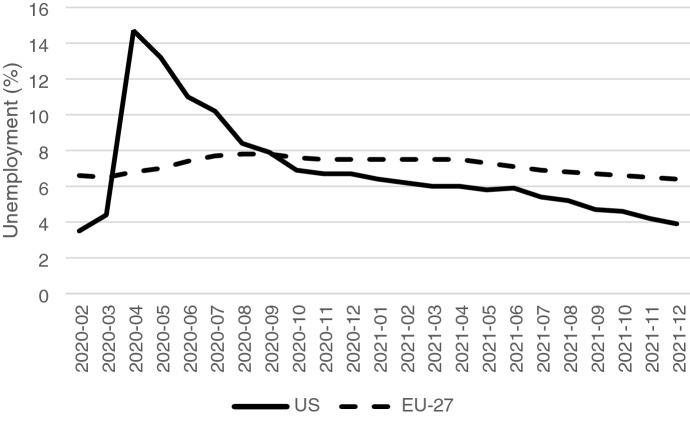
Monthly Unemployment Rate

## Appendix

### EU—Inter-State Polarisation over Recovery Funding

See Table 2.

**Table 2:** Partisan Affiliation of Member State Governments (‘Coronabond 9’ and Frugal 4 + 2 Blocs)

“Coronabond 9”	European Party Affiliation	Frugal Four + Germany, Finland
Italy—PM	Unaffiliated	–
Italy—Finance Spain Portugal	Party of European Socialists	Sweden Denmark Finland—PM Germany—Finance
Belgium—Finance	Alliance of Liberals and Democrats for Europe	Netherlands—PM
Belgium—PM France Ireland Luxembourg	Renew Europe	Finland—Finance
Greece Slovenia	European People’s Party	Netherlands—Finance Austria Germany—Chancellor

Source: Authors’ own elaboration

Finance Ministers listed when party different to Prime Minister (PM)/Chancellor

### EU—Italian Public Opinion on EU Membership

See Fig. 1.

### Functional Reference Indicators of Covid-19 Management (US/EU)

See Fig. 2, 3, 4 and 5.

## Footnotes

1 This paper is primarily concerned with political implications, but key metrics on functional pandemic policies in the EU and US are summarised in the "Appendix".

2 Rodden and Wibbels (2010) find that vertical party-political alignment leads to less fiscal freeriding by states.

3 For an anatomy of EU responses, see Rhodes (2021).

4 See Table 2, "Appendix" on positions and party groupings.
